# Eucalyptus in the Treatment of Scarlet Fever by Inunction

**Published:** 1893-06-03

**Authors:** Bertram Rogers


### NEW DRUGS AND PREPARATIONS.

[We should be glad to receive, at our office, 428, Strand, London, W.C., from the manufacturers, specimens of all new preparations and drugs which may be brought out from time to time.]

### EUCALYPTUS.

### IN THE TREATMENT OF SCARLET FEVER BY INUNCTION.

BERTRAM ROGERS, B.A., M.D.OXON.

That the infection of scarlet fever is contained in the desquamated particles of skin shed in the later stages of the disease there never has been any doubt, but authorities differ in their opinions of how long the virus continues to be got rid of in this manner. The practice that has usually been adopted to ensure the prevention of the spread of the disease is to keep the patient isolated until all desquamation has ceased, but we have seen it stated that, whether peeling or not, at the end of six weeks from the commencement of the illness the patient may without risk be discharged from isolation.

Numerous attempts have been made from time to time to render these desquamated particles aseptic, to kill the still undiscovered germ of scarlet fever, as it exists in these pieces of skin; but no one is so indiscreet as to allow a patient desquamating to mix with his fellow man, while trusting to inunction alone with our ordinary germ-killing reagents, for no doubt the infection is given off in other ways. To our mind—though, perhaps, our ideas may not be accepted by all medical men—we do not know what the germ is, whether a bacillus or micrococcus, spirillum or any other micro-organism; much less do we know its life history, or the reagents that kill it. Attempts have been made in the diseases which we know are caused by micro-organisms and the products formed by their growth, to destroy the germ by those reagents which laboratory experiments have proved are fatal to their growth, but we still treat phthisis, enteric fever, erysipelas, and many more, in the same way that they were treated in the days before the discovery of their respective micro-organisms. It is true an antidote for tetanus has been discovered, but as yet the time required to obtain this body is a bar to its general use.

The same obtains with scarlet fever. It is generally recommended in the text books, and we believe followed by medical men with that blind sheep-like simplicity which characterises our therapeutic practice—that the oil used for inunction should contain carbolic acid or some other antiseptic which has been found destructive to the anthrax bacillus in a test tube, forgetting that we do not know the micro-organism of scarlet fever, or whether it or its spores may or may not resist boiling in one in twenty carbolic for two hours. We argue, we say, from analogy that there is a germ of scarlet fever, and so employ carbolic oil, but there is not a particle of evidence to prove that the germ is killed by the antiseptic. All we know is that the oil prevents the light particles of shed skin from being blown about, and it is really to the oil that we trust for the prevention of the spread, and not the antiseptic reagent contained in it.

But we have recently in the February number of our contemporary, the *Medical Magazine*, had brought before us by Mr. Curgenven the results of inunction with a new preparation of eucalyptus. It is not our intention to review this interesting article, or to comment on the wonderful success obtained by Mr. Curgenven and other medical men, but to relate our own experiences of the use of Tucker's Oleusaban Eucalyptus Disinfectant, and to state how it failed to prevent the spread of scarlet fever which had broken out in a household under our care.

It may not be amiss to give an outline of Mr. Curgenven's method of treatment with this preparation, in case some of our readers may not have had the opportunity of consulting the article. As soon as the diagnosis of scarlet fever has been established, Mr. Curgenven recommends that the patient should be, so to speak, saturated with the disinfectant. This saturation is obtained by anointing the patient, and freely sprinkling the room and bed clothes with the eucalyptus preparation. This, efficiently done, will, he claims, render the patient so incapable of infecting a susceptible person that he has allowed his patient's younger sisters or brothers to remain in the room without their contracting the disease. He moreover claims that the course of the disease is averted or shortened, and complications are avoided. In some cases he administers the same preparation by the mouth, but that, we understand, is a work of supererogation.

The results obtained by Mr. Curgenven and the reports obtained from other medical men and schoolmasters with respect to the great value of this disinfectant in preventing epidemics inspired us with a desire to give it a fair trial on the earliest opportunity, and a case of scarlet fever presenting itself in a household containing five children and several servants, all susceptible, we forthwith ordered the preparation.

Our patient was a sickly girl of five. From where or whom she took the disease we have not been able to discover. No other member of the family took the disease, though on the first day of illness she, not liking the taste of a chlorate of potash tablet given her by her mother to relieve the sore throat, passed it on half-sucked to her brother. She was promptly isolated on the upper landing of the house, and for reasons that it is unnecessary to narrate, a susceptible nurse from the domestic servants was placed with her. The next day the services of a hospital nurse were obtained, and, as it was subsequently proved she was also susceptible to scarlet fever. Unfortunately for two days the particular preparation recommended by Mr. Curgenven was not forthcoming, so inunction and fumigation with the ordinary eucalyptus oil was commenced. On the fifth day of the girl's illness the hospital nurse complained of sore throat and next day presented all the symptoms of scarlet fever. She, like the child, was treated with Tucker's preparation, and both cases did well and recovered without any complication.

On the twenty-first day the domestic servant succumbed and passed through a mild course of the disease. In fact, the only person unprotected by a previous attack of scarlet fever who escaped the infection, was the medical man who now supplies his experiences of Tucker's Oleusaban Eucalyptus Disinfectant.

It might be objected that the want of the proper preparation in the early stages of the child's sickness was the cause of the nurse's taking the disease, but this cannot be claimed in the second case, unless we admit an unusually long incubation period, one for twenty days or so, a period unknown in scarlet fever. We did not give the eucalyptus internally; the nurse and child had biniodide of mercury, the domestic nurse a placebo, and in all cases the throat was sprayed with Sanitas; but we believe that Mr. Curgenven does not make the internal administration a *sine qua non* for success. All the other details, such as inunction and fumigation, &c., were carried out carefully and by skilled hands.

As regards the preparation itself, it certainly has a pleasant odour, the smell of the eucalyptus being more aromatic, and not so depressing in character as the well-known oil. It does not appear to be oily, or else in such a finely emulsified form that the sensation of oiliness is not felt. The smell is very persistent, and is not removed by several washings in water. All three patients complained of the smarting produced by the inunction, and when desquamation was free and tender skin exposed it was painful.

We do not consider that these cases are sufficient to condemn the use of this preparation, but failures as well as success in the use of new drugs should be recorded.

